# Leveraging multi-echo EPI to enhance BOLD sensitivity in task-based olfactory fMRI

**DOI:** 10.1162/imag_a_00362

**Published:** 2024-12-02

**Authors:** Ludwig Sichen Zhao, Clara U. Raithel, M. Dylan Tisdall, John A. Detre, Jay A. Gottfried

**Affiliations:** Department of Bioengineering, School of Engineering and Applied Science, University of Pennsylvania, Philadelphia, PA, United States; Department of Neurology, Perelman School of Medicine, University of Pennsylvania, Philadelphia, PA, United States; Department of Psychology, School of Arts and Sciences, University of Pennsylvania, Philadelphia, PA, United States; Department of Radiology, Perelman School of Medicine, University of Pennsylvania, Philadelphia, PA, United States

**Keywords:** multi-echo EPI, olfaction, ME-ICA, susceptibility artifacts, task-based fMRI

## Abstract

Functional magnetic resonance imaging (fMRI) usingblood-oxygenation-level-dependent (BOLD) contrast relies on gradient echoecho-planar imaging (GE-EPI) to quantify dynamic susceptibility changesassociated with the hemodynamic response to neural activity. However, acquiringBOLD fMRI in human olfactory regions is particularly challenging due to theirproximity to the sinuses where large susceptibility gradients induce magneticfield distortions. BOLD fMRI of the human olfactory system is furthercomplicated by respiratory artifacts that are highly correlated with eventonsets in olfactory tasks. Multi-echo EPI (ME-EPI) acquires gradient echo dataat multiple echo times (TEs) during a single acquisition and can leverage signalevolution over the multiple echo times to enhance BOLD sensitivity and reduceartifactual signal contributions. In the current study, we developed an ME-EPIacquisition protocol for olfactory task-based fMRI and demonstrated significantimprovement in BOLD signal sensitivity over conventional single-echo EPI(1E-EPI). The observed improvement arose from both an increase in BOLD signalchanges through aT_2_^*^-weightedecho combination and a reduction in non-BOLD artifacts through the applicationof the Multi-Echo Independent Components Analysis (ME-ICA) denoising method.This study represents one of the first direct comparisons between 1E-EPI andME-EPI in high-susceptibility regions and provides compelling evidence in favorof using ME-EPI for future task-based fMRI studies.

## 1 Introduction

Functional magnetic resonance imaging (fMRI) is one of the most common imagingtechniques used to non-invasively study both localized neural activation andwhole-brain functional connectivity in humans. Blood-oxygenation-level-dependent(BOLD) fMRI measures brain activity by detecting local magnetic susceptibilityfluctuations caused by changes in deoxyhemoglobin concentration. Gradient echoecho-planar imaging (GE-EPI) is the dominant BOLD imaging strategy due to its hightemporal signal-to-noise ratio and rapid acquisition capabilities (Budde et al., 2014). However, artifacts caused by thelocal inhomogeneous magnetic field surrounding high-susceptibility regions present amajor technical challenge in utilizing GE-EPI to measure BOLD signals. Olfactoryregions, including the orbitofrontal cortex (OFC), amygdala, piriform (olfactory)cortex, and entorhinal cortex, are particularly affected by susceptibility-inducedartifacts and signal drop-out due their location near the ethmoid sinuses(air-filled cavities in the bone surrounding the nose). These artifacts limit theapplicability of GE-EPI-based BOLD fMRI in pursuit of understanding the humanolfactory system (Catani et al., 2013).Moreover, respiratory artifacts, primarily caused by local field shifts duringinhalation and exhalation that are highly correlated with event onsets intasked-based olfactory-related fMRI, further compromise the sensitivity of olfactoryfMRI (Glover & Law, 2001).

Several techniques have been proposed to overcome these challenges. Acquiring scansat a tilted angle allows for partial compensation of local susceptibility gradients(Deichmann et al., 2003;Weiskopf et al., 2006). Alternatively, pulse sequencesother than GE-EPI, such as*T_2_*-Prepared BOLD fMRI andbalanced steady state free precession (bSSFP) (Miaoet al., 2021;Parrish et al.,2008), are employed.

Recent work on multi-echo EPI (ME-EPI), which acquires multiple gradient echoes ateach imaging timepoint, has shown potential to improve BOLD sensitivity andefficiency in resting-state fMRI (Gonzalez-Castilloet al., 2016;Lynch et al., 2020).In conventional single-echo EPI (1E-EPI), a single volume is acquired after each RFexcitation at a specific echo time (TE), which determines both image signalintensity as well as BOLD contrast (Donahue et al.,2011;Gati et al., 1997;van der Zwaag et al., 2009). At a shorter TE,the image is more proton-density weighted, has less BOLD contrast, and less severesignal dropout in regions with high local susceptibility gradients. Conversely, witha longer TE, the image becomes moreT_2_^*^-weighted, has greater BOLDcontrast, and more severe signal dropout. The BOLD contrast-to-noise ratio (CNR) isexpected to vary with TE, peaking at theT_2_^*^of each voxel (Donahue et al., 2011;Fera et al., 2004;Gati et al.,1997;Graham et al., 1996;Triantafyllou et al., 2011;van der Zwaag et al., 2009). However, various brainregions exhibit distinctT_2_^*^values,resulting in varying optimal TEs, making it impossible to optimize TE across thewhole brain in a single-echo acquisition.

Unlike 1E-EPI, ME-EPI acquires multiple images across a range of TEs after each RFexcitation, resulting in enhanced functional contrast by combining the echo imageswith different weights (Poser et al., 2006;Posse et al., 1999). The commonly usedT_2_^*^-weighted echo combinationmethod assigns the heaviest weight to the echo that is presumed to have the greatestBOLD CNR, with weighting performed separately for each voxel. By applying aT_2_^*^-weighted echo combinationwith ME-EPI, BOLD signal can be maximized at the voxel level while maintaining thetemporal resolution needed for fMRI. Moreover, by leveraging the expectedexponential signal decay across TEs for true BOLD contrast, noise reductiontechniques can be employed to reject artifactual signals and thereby improve theBOLD contrast-to-noise ratio (DuPre et al.,2021;Gonzalez-Castillo et al.,2016;Kundu et al., 2012,^2013^,^2017^;Van et al., 2023).

In this study, we implemented and tested an ME-EPI acquisition protocol and analysispipeline specifically designed for task-based olfactory-related paradigms byincorporating shorter TE values in an effort to improve sensitivity in ventral brainregions with high static susceptibility. Using this approach in comparison to astandard 1E-EPI acquisition, we demonstrate superior sensitivity to olfactory taskactivation, resulting in a significant sample size reduction for detecting grouptask activation during a representative olfactory three-alternatives forced choicetask (3AFC).

## 2 Method

### 2.1 Subjects

Twenty-nine subjects (13 women; aged 22–30 years, mean: 26, SD: 3.15)provided informed consent as approved by the University of PennsylvaniaInstitutional Review Board (#827217). One subject was excluded from the studydue to excessive motion (number of excluded volumes, as defined in StatisticalAnalysis, exceeded 2% of the number of total volumes) during fMRI scanningsession, leaving us with a final sample of*n*= 28. Allsubjects reported being right-handed nonsmokers with no history of significantneurological/psychiatric disorder or olfactory dysfunction.

### 2.2 Odor stimuli and delivery

Stimuli consisted of two odorants: lemon oil extract (12.3% v/v, Sigma-Aldrich,MO, USA) and benzaldehyde (0.42% v/v, Sigma-Aldrich, MO, USA). Both odorantswere diluted in mineral oil (Sigma Aldrich, MO, USA). Mineral oil alone was usedas a control stimulus. The odor stimuli were delivered through a custom-builtcomputer-controlled air-diluted 12-channel olfactometer with a flow rate of 4L/min controlled using the PsychToolbox package (Brainard, 1997) and MATLAB (Version 2020b, The Mathworks Inc.,Natick, MA, USA). Medical-grade air (Airgas, Radnor, PA, USA) was first routedthrough two computer-controlled 6-channel gradient valve manifolds (NResearchInc., West Caldwell, NJ, USA), regulated by a mass flow controller (MFC) (AlicatScientific Inc., Tucson, AZ, USA). Subsequently, it passed through the headspaceabove 2 mL of the mineral oil-diluted odorant before being delivered to theparticipant through a fluorinated ethylene-propylene (FEP) plastic tube (U.S.Plastic Corp., Lima, OH, USA) placed directly below the nose.

### 2.3 Behavioral testing session

**Fig. 1.**
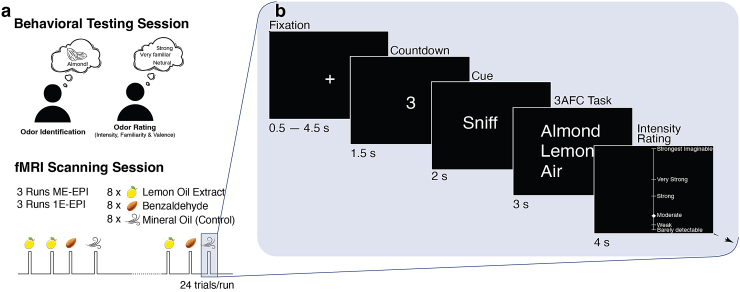
fMRI experimental paradigm*.*(a) The experiment wasconducted over the course of two sessions. On Day 0, the subjectsparticipated in a series of behavioral tests where they were required toidentify odors and rate their intensity, valence, and familiarity.Subsequently, an fMRI scanning session was scheduled for the subjects,taking place 2 to 5 days after their initial visit. This scanningsession consisted of three runs of ME-EPI and three runs of 1E-EPIacquisition. Each run comprised 24 trials, with an equal distribution ofthree odor stimuli: lemon oil extract, benzaldehyde, and mineral oil.(b) Timeline for a single trial. A trial started with a fixation crosspresented for 0.5–4.5 s, followed by a 1.5 s countdown. A cue tosniff was then displayed and the odor was delivered for 2 s. The shortodor onset and a long intertrial interval (11.5 s on average) werechosen to minimize habituation. Participants were then asked to identifythe odor and rate its intensity.

On Day 0, subjects participated in a behavioral testing session during which odorstimuli were presented outside the MRI environment (Fig. 1a). Participants were introduced to the odors andasked to label them as well as to rate the perceived odor intensity, via thegeneral Labeled Magnitude Scale (gLMS) (Green etal., 1993,^1996^). Valence(likeability) was also assessed via the Labeled Hedonic Scale (LHS) (L. V. Jones et al., 1955;Lim et al., 2009;Schutz & Cardello, 2001;Stone et al., 2020), as well as familiarity [Visual Analog Scale(VAS) (Dourado et al., 2021;Flint et al., 2000)]. Ratings were analyzedin MATLAB (Version 2020b, The Mathworks Inc., Natick, MA, USA), using threeone-way ANOVAs to evaluate whether there were any differences among stimuli.

### 2.4 fMRI scanning session

Two to 5 days after the behavioral testing session, the same participants tookpart in an fMRI scanning session, completing six runs, each of which contained24 trials. On each trial, participants sniffed one of the three possibleodorants (lemon oil extract, benzaldehyde, or mineral oil) and were asked toidentify the odor using a 3AFC task with the labels they provided earlier duringthe behavioral testing session. Following the 3AFC task, they were also asked torate the intensity of the odor. Each odor was presented eight times per run in arandom order. The experimental paradigm as well as the sequence of events withina given trial is shown inFigure 1.

### 2.5 Structural and functional MRI data acquisition

All magnetic resonance images were acquired using a 3 T scanner (MAGNETOM Prisma,Siemens Healthineers, Germany) using the vendor’s 64-channel head andneck coil.*T_1_*-weighted structural images wereacquired using ME-MPRAGE (TE = 1.69/3.55/5.41/7.27 ms, TR = 2530ms, TI = 1100 ms, FA = 8°, 1 mm isotropic).

1E-EPI and ME-EPI fMRI data were acquired using the sequence parameters outlinedinTable 1. For both protocols,repetition time (TR), field of view (FOV), number of slices, resolution, flipangle (FA), echo spacing, and multiband factors are identical. To optimize theBOLD signals in OFC regions, the field of view was tilted approximately25° to the anterior commissure-posterior commissure line (rostral> caudal) (Deichmann et al.,2003). The two sequence types were administered in an interleaved fashionin each participant, with the order being counterbalanced across participants.Of note, for 1E-EPI the echo time was set at 22 ms, which is approximately thesame as the second echo time out of five echoes in the ME-EPI sequence (seeTable 1). This TE aims to balancesignal dropout and BOLD sensitivity for olfactory-related regions and isconsistent with other olfactory-related fMRI studies (Bao et al., 2019;deGroot et al., 2021;Raithel et al.,2023;Shanahan et al., 2021;Zhou et al., 2019).

**Table 1.** fMRI sequence parameters.

	1E- EPI	ME-EPI
TR (ms)	2041	2041
TE (ms)	22	10.60/22.92/35.24/47.56/59.88
FOV (mm x mm)	216 x 216	216 x 216
Slices	48	48
Resolution (mm x mm x mm)	2.4 x 2.4 x 2.4	2.4 x 2.4 x 2.4
FA (degree)	78	78
Echo spacing (ms)	0.49	0.49
Bandwidth (Hz/Px)	2526	2646
Acceleration	Multiband 2x	Multiband 2x GRAPPA, 3x in-plane acceleration with 36 reference lines

TR: Repetition time; TE: Echo time; FOV: Field of view; FA: Flipangle; 1E-EPI: single-echo EPI; ME-EPI: multi-echo EPI.

Additionally, a pair of spin-echo EPI (SE-EPI) images with two phase-encode blipsare acquired for both fMRI acquisition protocols (1E-EPI and ME-EPI) separatelywith identical FOV, number of slices, resolution, FA, echo spacing,acceleration, and readout bandwidth compared to their respective fMRIacquisition. These pairs of images are then used to estimate inhomogeneities inthe static magnetic field (*B_0_*) during preprocessingusing a similar method described in (Andersson etal., 2003) and implemented in FSL (Smith et al., 2004) as part of the fMRIPrep package (Esteban et al., 2019;Gorgolewski et al., 2011).

### 2.6 fMRI data preprocessing

fMRI and structural MRI data were preprocessed in each subject’s nativespace using fMRIPrep 21.0.0 based on Nipype 1.6.1 (Esteban et al., 2019;Gorgolewski et al., 2011). All images were then registered to ICBM152 Nonlinear Asymmetrical template version 2009c (MNI-ICBM2009c) (2.4 mmisotropic resolution, consistent with fMRI acquisitions) before furtherprocessing (Fonov et al., 2009,^2011^). Spatial smoothing was applied to alldata using a Gaussian kernel of 8 mm full width at half maximum (FWHM). Adetailed description of these preprocessing steps can be found inSupplementary Materialsunder the Detailed fMRI Data Preprocessing section.

### 2.7. ME-EPI *T _2_* ^*^ estimation, echo combination, and multi-echo independent components analysis (ME-ICA)

Multi-echo EPI produces separate images for each echo time at each timepoint inthe functional imaging run. At each timepoint, these images for the multipleecho times were combined using theT_2_^*^-weighted combinationmethod (ME-WC) (Posse et al., 1999). Foreach voxel,T_2_^*^was estimated byfitting theEq. (1)using signalsfrom all five echoes,

$$S=S_{0}e^{-TE/T_{2}^{*}}$$ (1)

*S_0_*is the initial signal magnitude without anyT_2_^*^decay, and*S*is the signal magnitude acquired at*t*=*TE*.

The images from the five echoes are then combined voxel-wise using a weightedaverage (ME-WC), and the weights for each echo (*W*_TE_)are calculated usingEq. (2)based on estimatedT_2_^*^.

$$W_{TE}=\frac{TE\cdot e^{-TE/T_{2}^{*}}}{\sum_{n=1}^{5}TE_{n}\cdot e^{-TE_{n}/T_{2}^{*}}}$$ (2)

Independent components analysis (ICA) is a common strategy to decompose fMRIsignals into distinct independent components, allowing the isolation ofBOLD-related neuronal components from sources of noise (Beckmann, 2012;Caballero-Gaudes & Reynolds, 2017;Mckeown et al., 1998,^2003^). However, a persistent challenge lies in the manualclassification of these independent components, which is labor-intensive and canyield inconsistent results (Kelly et al.,2010). In an attempt to tackle this problem, ME-ICA classifies eachcomponent as either a non-BOLD artifact, such as respiratory artifact, or aBOLD-like signal based on their signal characteristics across different echotimes (Kundu et al., 2012,^2013^). Here, we specifically employed theTE-dependency analysis pipeline (Tedana 0.0.12) (DuPre et al., 2021) to preprocess multi-echo EPI data beforeregistering them to the MNI template.

In the original TEDANA pipeline, a monoexponential model [Eq. (1)] was fit to the data ateach voxel using nonlinear model fitting to estimate*T*_2_^*^and*S_0_*maps, using*T*_2_^*^/*S_0_*estimates from a log-linear fit as initial values. This approach generallyperforms well across most brain regions. However, in regions with significantsignal dropout at longer echo times, particularly due to susceptibilityartifacts in areas such as the orbitofrontal cortex and entorhinal cortex, thisfitting method tends to overestimate*T*_2_^*^. Consequently, we replacedthe TEDANA estimate ofT_2_* with our ownestimates, generated by fitting the following model voxel-wise:

$$S=\sqrt{{\left(S_{0}e^{-TE/T_{2}^{*}}\right)}^{2}+x_{0}^{2}}$$ (3)

In this model,*x_0_*represents the thermal noise floor,which does not decay over TE and whose contribution to the overall signalincreases in later echoes as the signal diminishes in high-susceptibilityregions. This model was fit to the data by minimizing the 2-norm of the weightedresiduals [Eq. (4)] via theTrust-Region-Reflective algorithm (Coleman& Li, 1996).

$$r_{i}=\frac{S_{i}}{\sum_{j=1}^{n}S_{j}}\left(\sqrt{{\left(S_{0}e^{-TE_{i}/T_{2}^{*}}\right)}^{2}+x_{0}^{2}}-S_{i}\right)$$ (4)

The weighted residuals are calculated as the difference between the estimatedsignals${\hat{S}}_{i}$and the observed signals$S_{i}$,weighted by the observed signals$S_{i}$across all*n*= 1000 observations (200 volumes/run, 5echoes/volume).

### 2.8 Estimating motion for different odor stimuli

The framewise displacement (*FD*), calculated during fMRIpreprocessing using the method described in (Power et al., 2012), and the onset of odor stimulus for eachcondition were extracted for each run. The coefficient of determination(*R^2^*) was then estimated by calculating thePearson correlation coefficient between the*FD*and the odoronset for each stimulus condition convolved with the canonical hemodynamicresponse function (HRF) for each subject (Pearson, 1895;Siegel, 1956).To compare whether there is a group-level difference in movement parameters fordifferent odor stimuli, two-sided paired*t*-tests were performedon the*R^2^*, across all three stimulus conditions([Lemon vs. Control], [Benzaldehyde vs. Control], and [Lemon vs. Benzaldehyde])for both 1E-EPI and ME-EPI acquisitions.

### 2.9 Physiological data analysis

The respiratory trace was recorded with a respiratory transducer (breathing belt)using a pressure sensor (BIOPAC System, CA, USA) affixed around thesubject’s chest, and then preprocessed before being added as a nuisanceregressor: the trace was smoothed using a moving average with a 250 ms window,high-pass filtered at 0.05 Hz with an infinite impulse response (IIR) filter,detrended, and standardized such that the mean of the signals is 0 and thestandard deviation is 1 (Liu et al.,2024;Raithel et al., 2023). Thepreprocessed trace was also downsampled to 1/2.4 Hz to match the*TR*of the fMRI acquisitions and included as a nuisanceregressor. Two additional nuisance regressors were introduced: thetrial-by-trial sniff volume was defined as the amplitude difference between thepeak and the trough within the cued sniff, and the sniff duration was defined asthe time difference between the peak and the trough.

### 2.10 Statistical analysis

All fMRI data were analyzed in SPM12 using a general linear model (GLM) toestimate the main effects of the odor stimulation. Each experimental condition(control, lemon oil, and benzaldehyde) was modeled using an independentregressor for each run. This results in a 2 (multi-echo and single-echo) by 3(control, lemon oil, and benzaldehyde) factorial design. The model included thefollowing nuisance regressors: trial-by-trial sniff volume and sniff duration,both convolved with the hemodynamic response function (HRF), as well as thedown-sampled breathing trace and 24 movement parameters (six movement parametersderived from spatial realignment, their squares, derivatives, and squares ofderivatives). Additional nuisance regressors were introduced to exclude volumeswith excessive motion, defined as those with at least one of six movementparameters derived from spatial realignment deviating by more than 6 standarddeviations from the mean. In all GLMs, data were high-pass filtered at 1/128 Hz;the temporal autocorrelation was estimated and removed as an autoregressiveAR(1) process globally (Friston et al.,2002).

Contrasts of interest were defined as [Odor > Control], [Lemon >Control] and [Benzaldehyde > Control]. Whole-brain analysis was performedto validate that odor-evoked activations were present in the data acquiredthrough both protocols (1E-EPI and ME-EPI). Small volume corrections (SVC) andROI analysis were separately performed on OFC, amygdala, piriform cortex, andentorhinal cortex, as suggested by previous studies highlighting the involvementof these regions in human olfactory processing (Anderson et al., 2003). Amygdala and piriform cortex were definedanatomically using a human brain atlas (Mai etal., 2016). Delineation of OFC and entorhinal cortex was based on theMNI-ICBM2009c atlas (Manera et al.,2020).

### 2.11 Post-hoc power analysis

We used our ROI analysis results to estimate the sample sizes required to detectsignificant olfactory-related group fMRI activation in subsequent studies. Apriori power analyses were performed with*α*=0.05 and*β*= 0.80 using G*Power (Faul et al., 2009) for both ME-EPI and1E-EPI data in the same ROIs described in the previous section.

## 3 Results

### 3.1 Echo-combined images reduce signal dropout

**Fig. 2.**
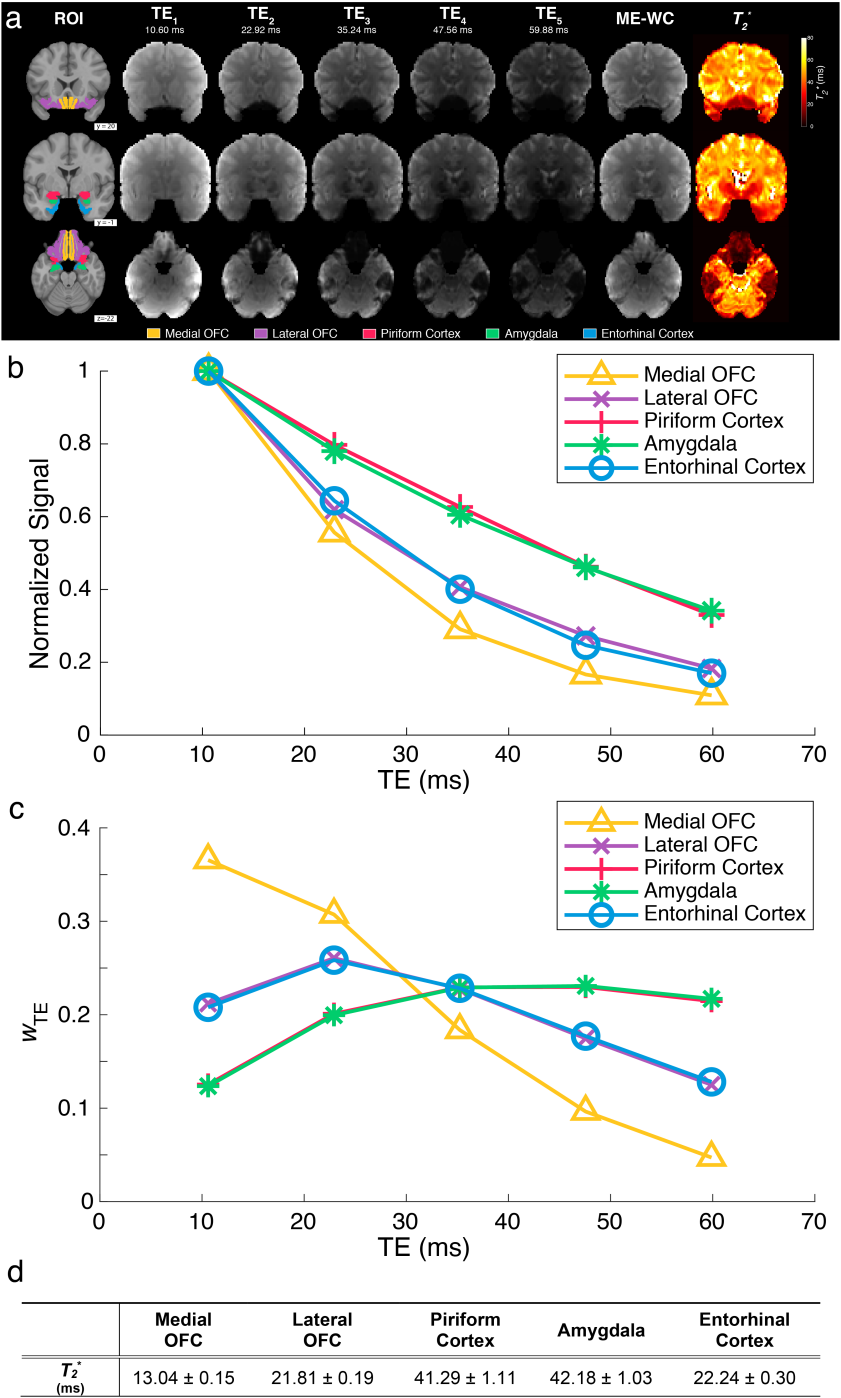
Multi-echo fMRI reduces signal dropout and improves BOLD contrast throughT_2_^*^-weightedcombination in a single subject*.*(a) Two representativecoronal slices containing the regions of interest are shown with avariety of contrasts: ROIs overlaid on the*T_1_*-weighted image (ROI); estimatedT_2_^*^maps(T_2_^*^); all fiveindividual echo images (TE_1_–TE_5_) from theME-EPI acquisition; and theT_2_^*^-weighted combinedimages (ME-WC). (b) Plot of signal versus echo time for each anatomicalROI within a single subject. Within each ROI, mean signals for eachindividual echo were normalized by dividing by the mean signal from thefirst echo. (c) Within-ROI mean of the voxel-wise weights used tocompute theT_2_^*^-weightedcombined images for the same subject. (d) Estimated meanT_2_^*^± 95%confidence interval for each ROI.

We first examined the estimatedT_2_^*^values for each olfactory-related region. As expected, signal dropouts arereduced in shorter echo times (Fig. 2a,TE_1_and TE_2_), whileT_2_^*^-weighted contrast isgreater in later echo times (Fig. 2a,TE_3_–TE_5_). In particular, the medial and lateralOFC along with the entorhinal cortex exhibited notably smallerT_2_^*^values due tosusceptibility artifacts (Fig. 2a &2b), and in turn the echo-combined image more heavily weights theshorter echoes (Fig. 2c). Using theseweights, the combined image (Fig. 2a,ME-WC) effectively mitigated signal dropout while retaining notableT_2_^*^-weighted contrast.

### 3.2 BOLD activation in diverse olfactory brain regions during odor stimulation

**Fig. 3.**
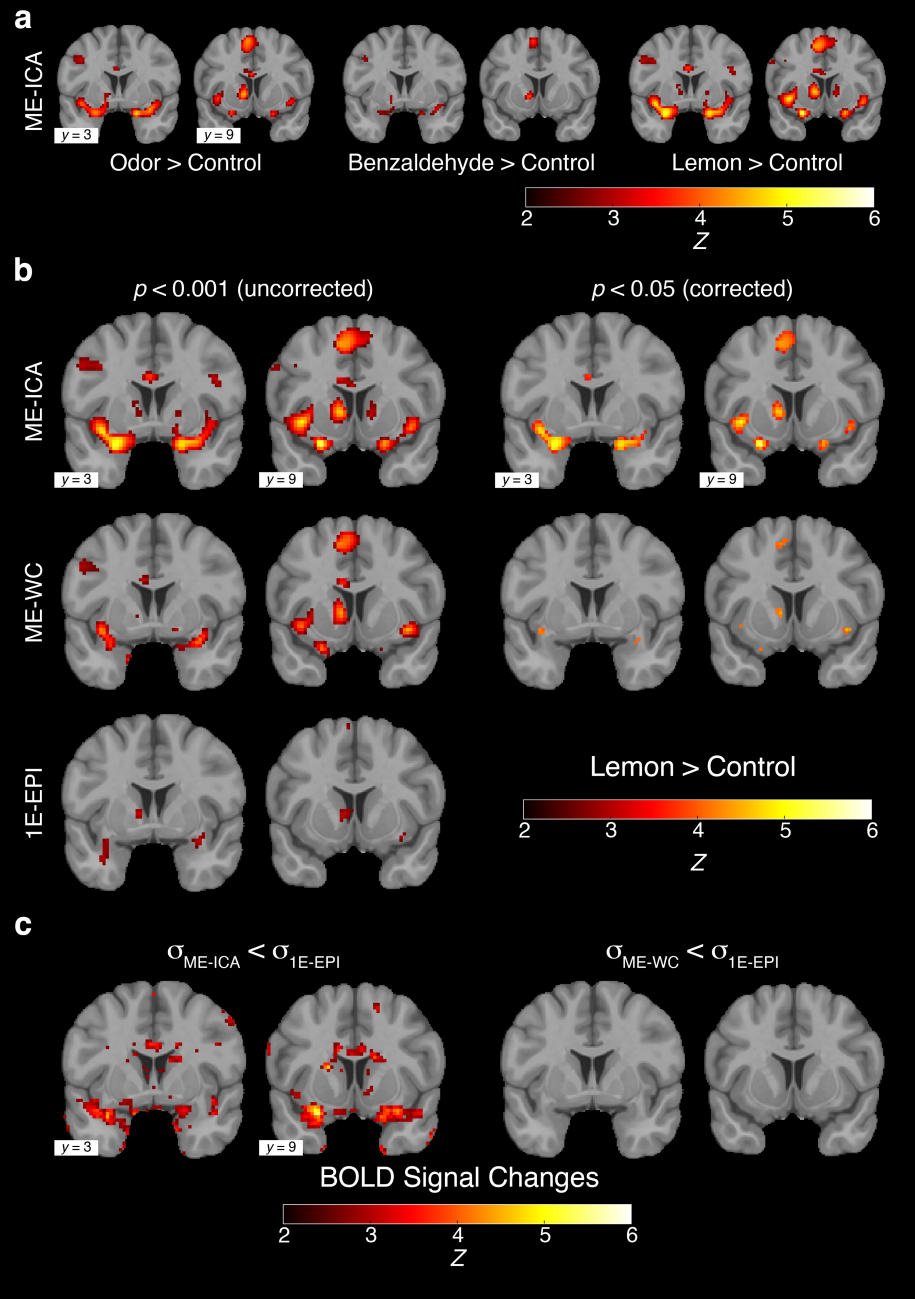
Activation maps from the 3AFC olfactory task for both 1E-EPI and ME-EPI.(a) Two representative coronal slices of activation maps for all threecontrasts ([Odor > Control], [Benzaldehyde > Control],[Lemon > Control]) using ME-EPI acquisition with ME-ICA denoisingmethods (*p*< 0.05, FDR corrected). (b)Activation maps for [Lemon > Control] contrast for ME-EPIacquisition with ME-ICA denoising methods (ME-ICA), and without ME-ICAdenoising methods (ME-WC) as well as single-echo fMRI acquisition(1E-EPI). The left two columns show the maps without multiple comparisoncorrection (*p*< 0.001), while the right twocolumns show the maps corrected for multiple comparisons(*p*< 0.05, FDR corrected). No activationsurvived correction for multiple comparisons for 1E-EPI (not shown). (c)*Z*-score statistical maps yielded fromLevene’s test to examine whether BOLD signal variance was lowerin ME-WC [σ_ME-WC_< σ_1E-EPI_]and ME-EPI [σ_ME-ICA_<σ_1E-EPI_] compared to 1E-EPI (*p*< 0.05, FDR corrected).

We next investigated the regions that were significantly activated when the odorswere delivered under ME-EPI acquisition with the ME-ICA denoising method [Odor> Control] (Fig. 3a) (DuPre et al., 2021). Consistent withprevious literature, we observed significant activation in the piriform cortex,amygdala, entorhinal cortex, insula, and OFC bilaterally (*p*< 0.001, uncorrected) (Anderson et al.,2003;Dikecligil & Gottfried,2024;Gottfried et al., 2003;Howard & Gottfried, 2014;Patin & Pause, 2015;Plailly et al., 2008). We next examined theBOLD effects of individual odor stimuli, namely [Benzaldehyde > Control]and [Lemon > Control]. Despite statistical tests indicating nosignificant differences in perceived intensity (*p*=0.38), valence (*p*= 0.45), and familiarity(*p*= 0.11) between benzaldehyde and lemon oil and nosignificant differences in motion among three experimental conditions ([Lemonvs. Control]*p*= 0.60, [Benzaldehyde vs. Control]*p*= 0.33, and [Lemon vs. Benzaldehyde]*p*= 0.63), our analysis revealed that the maineffect [Odor > Control] was driven mainly by the contrast [Lemon >Control]. In comparison, the contrast [Benzaldehyde > Control] producedsignificant levels of activation only in the bilateral insular region (Fig. 3a).

The difference in BOLD response to these stimuli was not anticipated though notwholly unprecedented, as previous research has demonstrated that different odorscan elicit distinct responses within olfactory brain regions even in the absenceof differences in low-level stimulus features (Fournel et al., 2016;Howard& Gottfried, 2014;Savic,2002;Savic et al., 2002). Inthe single-echo fMRI, a similar trend is observed, with more active regionsbeing detected with lemon oil (*p*< 0.05, uncorrected,Fig. S3). To ourknowledge, there is no literature in humans that directly elicits piriformcortex BOLD activation with a similar sample size for benzaldehyde. However,rodent studies have reported that benzaldehyde demonstrates BOLD activation inthe amygdala, as well as the piriform and entorhinal cortices (Kulkarni et al., 2012). Accordingly, to compare andobserve the differences between acquisition methods within olfactory regions, wewill focus on the activation under lemon oil condition [Lemon > Control]for our further analyses.

### 3.3 ME-EPI improves fMRI signal detection by both increasing BOLD signal sensitivity and specificity

Having validated that our ME-EPI acquisition protocol is suitable to captureodor-evoked BOLD responses, we next compared BOLD activation among differentacquisition methods (1E-EPI vs. ME-EPI) and analysis pipelines (ME-WC vs.ME-ICA). A whole-brain analysis using either whole-brain multiple comparisonscorrection with false discovery rate (FDR) (Benjamini & Hochberg, 1995;Benjamini & Yekutieli, 2001;Yekutieli & Benjamini, 1999) or small volume correction (SVC)did not yield any supra-threshold voxels for 1E-EPI. However, the samewhole-brain analysis using theT_2_^*^-weighted combined ME-EPI(ME-WC) highlighted activation in all ROIs (piriform cortex, amygdala, OFC, andentorhinal cortex) (Fig. 3b). Moreover, byusing ME-ICA denoising methods (DuPre et al.,2021), we observed a further increase in both the number ofactivation voxels and peak*Z*values in all ROIs (Table 2).

**Table 2.** Number of voxels and peak*Z*-score activated by lemon oilfor each ROI (*p*< 0.05, SVC).

	Piriform cortex			Amygdala		Entorhinal cortex		OFC
	# Voxel	Peak *Z*	# Voxel	Peak *Z*	# Voxel	Peak *Z*	# Voxel	Peak *Z*
ME-ICA	158	5.43	6	4.21	11	4.78	27	5.43
ME-WC	4	4.32	2	3.63	1	3.82	20	4.62
1E-EPI	0	N/A	0	N/A	0	N/A	0	N/A

We next examined potential reasons for the observed differences. Specifically, wehypothesized the improvement of the BOLD sensitivity in the case of the ME-EPIdata was due to larger BOLD signal changes (i.e., greater sensitivity), as weperformed a locally weighted combination of all echoes for each individual voxelinstead of using one single TE across the brain. BOLD signal changes could becalculated by contrast estimates from the GLM. Therefore, we performed a paired*t*-test on contrast estimates [Lemon > Control]between 1E- and ME-EPI with ME-ICA [ME-ICA > 1E-EPI] among all ROIs. Onlypiriform cortex (*p*= 0.023) and lateral OFC(*p*= 0.006) showed greater estimates for thecontrast [Lemon > Control] for ME-ICA compared to 1E-EPI.

An alternative explanation for the observed increase in BOLD detection for ME-EPIversus 1E-EPI may lie in the reduction of the background noise due to non-BOLDsignals (i.e., greater specificity). Accordingly, the signal acquired withME-EPI exhibits less fluctuation over time, resulting in lower variance and, inturn, increasing*Z*-scores. To test the hypothesis that ME-EPIsignals have lower variance, we performed a Levene’s test (Levene, 1960). This test is commonlyemployed to assess whether two or more groups have equal variances in a dataset(Chiarello et al., 2009;Farinha et al., 2022;Im et al., 2010;T.B. Jones et al., 2010;Swarup et al.,2013). When using a whole-brain analysis to compare the signalvariance between ME-ICA and 1E-EPI, we observed suprathreshold clusters(*p*< 0.05, FDR corrected) overlapping with thevoxels activated for the [Lemon > Control] contrast (*p*< 0.05, FDR corrected) from ME-ICA (Fig.3c). We then generated a mask using those activated voxels and foundthat 18.89% of the masked voxels reached the significance threshold underLevene’s test. This suggests that ME-ICA decreases the signal variance,thereby reducing non-BOLD noise and increasing BOLD specificity. We did notobserve any suprathreshold clusters when comparing ME-WC and 1E-EPI.

Taken together, these tests suggest that our observed increase in BOLD detectionis not only due to increased sensitivity to BOLD signals, but also the reductionof non-BOLD artifacts enhancing BOLD specificity.

### 3.4 ME-EPI improves statistical power and reduces sample sizes

**Fig. 4.**
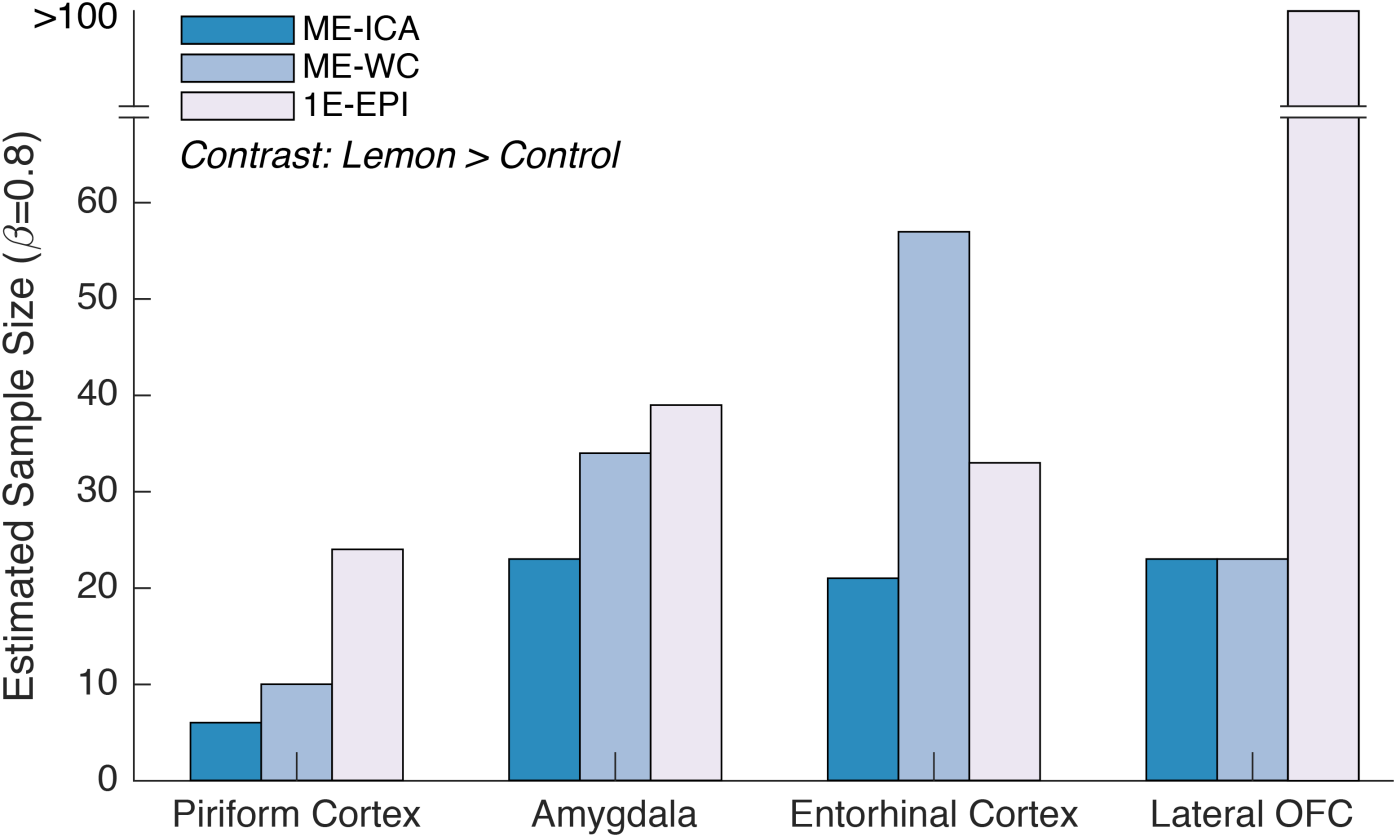
Estimated sample size for ROI analyses based on post-hoc power analyses(α = 0.05 and β = 0.80) in allolfactory-related ROIs under contrast [Lemon > Control] forsingle-echo fMRI (1E-EPI), ME-EPI BOLD acquisition with ME-ICA denoisingmethods (ME-ICA), and without ME-ICA denoising methods (ME-WC). Theestimated sample sizes for medial OFC for 1E-EPI, ME-ICA, and ME-WC werenot shown since they were all above 100.

So far, we have established that ME-EPI improved BOLD signal sensitivity by bothenhancing BOLD signal and reducing non-BOLD artifacts. An outstanding questionis to what degree BOLD sensitivity is improved when using our suggested methodin the context of task-based olfactory fMRI. To quantify the change in BOLDsensitivity, we examined the contrast [Lemon > Control] in the brainregions central to olfaction, namely the piriform cortex, amygdala, lateral andmedial OFC, and entorhinal cortex for both acquisition methods separately andthen compared the statistical outcomes. Whereas activation in medial OFC did notreach statistical significance using data resulting from either acquisitionmethod, all remaining regions show much lower*p*-values forME-ICA compared to 1E-EPI (Table 3).Furthermore, to determine the minimum sample size required to observesignificance in each region, post-hoc power analyses were carried out(*α*= 0.05 and*β*= 0.80) to estimate the effect of each acquisition method on the samplesizes needed to detect effects in similar olfactory task fMRI studies. ME-ICAled to a substantial reduction in the sample size, ranging from by half toone-fourth, compared to 1E-EPI, depending on the specific regions (Fig. 4).

**Table 3.** Statistical significance of ROI analyses for each olfactory-related ROIunder the contrast [Lemon > Control] for 1E-EPI, ME-WC, andME-ICA.

	Piriform cortex	Amygdala	Entorhinal cortex	Lateral OFC	Medial OFC
ME-ICA	<0.0001***	0.0052**	0.0039**	0.0058**	0.7663
ME-WC	0.0001***	0.0173*	0.0505	0.0054**	0.5607
1E-EPI	0.0066**	0.0243*	0.0157*	0.5429	0.6687

***p< 0.001,**p< 0.01, *p< 0.05.

## 4 Discussion

Susceptibility artifacts pose a significant challenge when using GE-EPI for fMRIstudies. The primary olfactory cortex not only suffers from some of the most severestatic susceptibility artifacts within the human brain but is also affected byrespiration-related artifacts. Here, we have developed and validated an ME-EPIprotocol and analysis pipeline specifically tailored to olfactory task-based fMRI.In the context of a basic olfactory 3AFC task, we found that ME-EPI, alongsideME-ICA denoising, systemically improves BOLD sensitivity and reduces the requiredsample sizes needed to infer a significant effect at a group level compared toconventional 1E-EPI. This is achieved by both increasing BOLD signal sensitivity andreducing the contributions of non-BOLD signal changes using the additional denoisingfeatures ME-EPI provided.

The echo times used for ME-BOLD provided a range of contrasts across different TEsfor each ROI of interest (Fig. 2a). Whenevaluating theT_2_^*^-derived weightscalculated for the echo-combined image, we confirmed that high-susceptibilityregions with shortT_2_^*^values, namely,lateral and medial OFC, and entorhinal cortex, had higher weights assigned to thefirst two echoes. In contrast, the remaining ROIs had higher weights assigned to theremaining (and later) three echoes. As a result, theT_2_^*^-weighted combined (ME-WC)images were able to produce more uniformT_2_^*^-weighted contrast across thewhole brain (Fig. 2a, ME-WC).

When investigating the region activated by odor onset using ME-ICA, we detectedactivation in all major primary olfactory cortex regions (piriform cortex, amygdala,entorhinal cortex, and OFC bilaterally), in addition to the insular region (Fig. 3a). These findings are consistent withprevious literature (Anderson et al., 2003;Dikecligil & Gottfried, 2024;Gottfried et al., 2003;Howard & Gottfried, 2014;Patin & Pause, 2015;Plailly et al., 2008). Further analysis revealed that theactivation was primarily driven by the lemon extract stimulus in comparison tobenzaldehyde. Previous studies have demonstrated that different odor stimuli canactivate distinct primary olfactory regions to different degrees (Fournel et al., 2016;Howard & Gottfried, 2014;Savic,2002;Savic et al., 2002). In thisstudy, we found that that benzaldehyde activates the olfactory system to a lesserextent than lemon oil, such that the observed activation did not reach significanceafter correcting for multiple comparisons in the whole-brain analysis.

When comparing the results of 1E-EPI and ME-EPI, we observed significantly increasedsensitivity in both whole-brain analysis and ROI analysis, which are two commonlyused univariate fMRI analysis methods. In the case of whole-brain analysis (Fig. 3b), we found that ME-EPI exhibited higherpeak*Z*values and a greater number of activated voxels compared to1E-EPI, as shown inTable 2. It is worthnoting that even without using the ME-ICA denoising method, ME-EPI stilloutperformed 1E-EPI by performing aT_2_^*^-weighted combination of datafrom all echoes. This improvement was expected, as the weighting of data from eachecho time was designed to enhance the sensitivity to BOLD contrast (Posse et al., 1999).

For ROI analysis and sequential power analysis, which were used to estimate minimalsample sizes, ME-EPI with the ICA denoising method demonstrated superiority over1E-EPI, as shown inTable 3andFigure 4. We attribute these improvements to twofactors: the increase of BOLD signal intensity throughT_2_^*^-weighted echo combinationand the reduction of noise by leveraging signal evaluation across the echo times. Tosupport these hypotheses, we conducted a series of statistical tests (Fig. 3c) to show the mean signals aresignificantly higher and the variance is significantly lower in ME-ICA acquisitioncompared to 1E-EPI.

While both ME-EPI and ME-WC overwhelmingly increased the statistical power for ROIanalysis, we observed an unexpected decrease in statistical power for ME-WC in theentorhinal cortex. Previous studies have demonstrated that local*B_0_*field shifts affectT_2_^*^values near the ethmoidsinuses during respiration, resulting in fluctuations ofT_2_^*^values across respiratorycycles (Barry & Menon, 2005;Raj et al., 2000,^2001^;Van de Moortele etal., 2002). TheT_2_^*^-weighted combination method usedin our analyses adopted a fixed weighting approach throughout a single scan,potentially failing to account for these dynamicT_2_^*^fluctuations. However, ouradditional analyses showed that, although statistically significant in most ROIs,T_2_^*^values exhibit only subtledifferences between inhalation and exhalation phases (Table S1). This, in return,demonstrates the robustness of the echo combination method against respiratory phasevariations.

We also evaluated whether nonexponential signal decay contributed to suboptimal echocombination weightings, leading to the unexpected result. As shown inFigure S2, signals frommost regions fit well with exponential decay, but regions with high susceptibility,such as the entorhinal cortex, medial, and lateral OFC, exhibited more pronouncednonexponential decay. Yet, this observation does not fully explain the unexpecteddecreases observed exclusively in the entorhinal cortex.

Furthermore, we examined the effects of ME-WC and ME-ICA on the distribution ofstatistical significance within each ROI (Fig. S1). We have shown that ME-WC increases sensitivity toboth positively correlated BOLD activation and anti-correlated signals. Moreover,ME-WC and ME-ICA both increase the peak*Z*-scores and the number ofstatistically significant voxels, consistent with voxel-wise whole-brain analysis(Table 2andFig. 3b).

Additionally, we investigated the effect of the number of echoes on the main effect,considering whether later echoes contribute significantly. Using the same analysispipeline, we compared whole-brain analysis results from data including the first 3,4, and all 5 echoes (Fig.S4). As the number of echoes increased, the size of the clusters alsoincreased.

The current analysis pipeline utilized a specific method of combining multiple echoimage series into a single image series:T_2_^*^-weighted combination, alsoknown as “optimal combination”, is used in most ME-EPI studies as itis designed to maximize BOLD-contrast sensitivity (Baracchini et al., 2021;Gonzalez-Castillo et al., 2016;Kundu etal., 2017;Lynch et al., 2020;Roth et al., 2020). However, aninteresting finding emerged when we processed the ME-EPI data separately and treatedeach individual echo series as a 1E-EPI dataset. Surprisingly, no supra-thresholdactivation was detected at the first echo (*p*< 0.05,uncorrected), while the majority of activations were observed at the later echoes(*p*< 0.05, uncorrected) (Fig. S5). When examiningthe*Z*values for the ROI analyses across all 5 individual echoes,we also observe a similar trend (Table S2). However, regions with shortT_2_^*^(such as the lateral andmedial OFC and entorhinal cortex in this study) predominantly assigned higherweights to the first two echoes.

Interestingly, in the process of understanding theT_2_^*^-weighted combinationmethod implemented in the TEDANA package, we observed that directly estimatingT_2_^*^by fitting theEq. (1)tends to overestimateT_2_^*^in regions with highsusceptibility (TableS3). As described in the Method, we proposed usingEq. (3)as the objective function toestimateT_2_^*^for each voxel. Whilethis approach improved some estimations, work is needed to improve the accuracy ofthese estimations in the future.

There have also been recent advances in denoising methods that leverage phaseinformation, such as Noise Reduction with Distribution Corrected (NORDIC) PCA (Dowdle et al., 2023;Moeller et al., 2021;Vizioli et al., 2021). These methods have demonstrated the ability tofurther enhance BOLD sensitivity in resting-state fMRI. However, our study did notacquire phase data, which prevented us from testing these advanced denoising methodsin our data.

In this study, we utilized a limited set of two odor stimuli, along with a controlstimulus. Although this approach was adequate to compare the ME-EPI and 1E-EPIacquisition methods, future studies will aim to incorporate a wider range of odorstimuli. By including multiple odor stimuli, we can expand our analysis to encompassmultivariate analysis, such as Multivariate Pattern Analysis (MVPA), which has beencommonly utilized in other olfaction task-based fMRI studies (Donoshita et al., 2021;Howard et al., 2009;Howard &Gottfried, 2014;Perszyk et al.,2023), though we expect also to see an improvement in multivariateanalysis using ME-ICA.

In conclusion, our study demonstrated the utility of an ME-EPI protocol combined witha pre-configured analysis pipeline for fMRI in olfactory cortex. This approachenhanced sensitivity to task-dependent BOLD contrast changes in olfactory regions,and post-hoc power analyses indicate that group effects can be detected with areduced sample size compared to standard 1E-EPI. Although our protocol and pipelinewere specifically designed for task-based fMRI in the presence of large staticsusceptibility, a longstanding challenge in the field of fMRI, they showcase thesuperior denoising capabilities of ME-EPI for task-based fMRI more generally. Thesefindings strongly support the adoption of ME-EPI over 1E-EPI for future task-basedfMRI studies.

## Supplementary Material

Supplementary Material

## Data Availability

The unthresholded*Z*-maps showing the whole-brain results forunivariate analyses were made available on Neurovault (https://neurovault.org/collections/KGZMOMWR/). The modified TEDANA codeswith updated*T_2_** estimation methods described inSection 2.7are available on GitHub (https://github.com/luxwig/tedana/tree/altT2s).
