# Correction to: Leveraging multi-echo EPI to enhance BOLD sensitivity in task-based olfactory fMRI

**DOI:** 10.1162/imag_x_00421

**Published:** 2024-12-18

**Authors:** Ludwig Sichen Zhao, Clara U. Raithel, M. Dylan Tisdall, John A. Detre, Jay A. Gottfried

**Affiliations:** Department of Bioengineering, School of Engineering and Applied Science, University of Pennsylvania, Philadelphia, PA, United States; Department of Neurology, Perelman School of Medicine, University of Pennsylvania, Philadelphia, PA, United States; Department of Psychology, School of Arts and Sciences, University of Pennsylvania, Philadelphia, PA, United States; Department of Radiology, Perelman School of Medicine, University of Pennsylvania, Philadelphia, PA, United States

The columns inTable 2were previously misgrouped and have been corrected here. The table contents, including data and captions, remain unchanged.

**Table 2.** Number of voxels and peak*Z*-score activated by lemon oil for each ROI (*p*< 0.05, SVC).

	Piriform cortex		Amygdala		Entorhinal cortex		OFC	
	# Voxel	Peak *Z*	# Voxel	Peak *Z*	# Voxel	Peak *Z*	# Voxel	Peak *Z*
ME-ICA	158	5.43	6	4.21	11	4.78	27	5.43
ME-WC	4	4.32	2	3.63	1	3.82	20	4.62
1E-EPI	0	N/A	0	N/A	0	N/A	0	N/A

