# Supplementary material for: Leveraging multi-echo EPI to enhance BOLD sensitivity in task-based olfactory fMRI
Source: Imaging Neurosci (Camb). 2024 Dec 2;2:imag-2-00362. doi: 10.1162/imag_a_00362 (PMC12315737; doi:10.1162/imag_a_00362)
Supplement: Supplementary Material [file imag_a_00362-supp.pdf]

Supplementary Materials

Leveraging Multi-Echo EPI to Enhance BOLD Sensitivity in Task-based Olfactory fMRI

Ludwig Sichen Zhao[*1,2], Clara U. Raithel[3,2], M. Dylan Tisdall[4], John A. Detre[2,4], and Jay A. Gottfried[*2,3]

[1] *Department of Bioengineering, School of Engineering and Applied Science, University of Pennsylvania, Philadelphia, PA, United States*
[2] *Department of Neurology, Perelman School of Medicine, University of Pennsylvania, Philadelphia, PA, United States*
[3] *Department of Psychology, School of Arts and Sciences, University of Pennsylvania, Philadelphia, PA, United States*
[4] *Department of Radiology, Perelman School of Medicine, University of Pennsylvania, Philadelphia, PA, United States*

* Corresponding Author: Ludwig Sichen Zhao (ludwigz@seas.upenn.edu), Jay A. Gottfried (jaygottf@pennmedicine.upenn.edu)

Table of Contents

1 Detailed fMRI Data Preprocessing 1
1.1 Preprocessing of B_0 Inhomogeneity Mappings 1
1.2 Anatomical Data Preprocessing 1
1.3 Functional Data Preprocessing 2
2 Impacts of ME-WC and ME-ICA on the Distribution of Statistical Significance 5
3 T_2^* Changes Due to Respiration 7
4 Nonexponential Decay of Multi-Echo GE-EPI Signal 8
5 Supplementary Figures and Tables 10
6 Supplementary References 14

1 Detailed fMRI Data Preprocessing

Results included in this manuscript come from preprocessing performed using *fMRIPrep* 21.0.0 (Esteban, Markiewicz, et al. (2018); Esteban, Blair, et al. (2018); RRID:SCR_016216), which is based on *Nipype* 1.6.1 (K. Gorgolewski et al. (2011); K. J. Gorgolewski et al. (2018); RRID:SCR_002502).

1.1 Preprocessing of B_0 Inhomogeneity Mappings

A total of 2 fieldmaps were found available within the input BIDS structure for this particular subject. A B_0-nonuniformity map (or *fieldmap*) was estimated based on two (or more) echo-planar imaging (EPI) references with topup (Andersson, Skare, and Ashburner (2003); FSL 6.0.5.1:57b01774). Briefly, the 2 fieldmaps were collected with reversed phase-encode blips (anterior to posterior and posterior to anterior) as described in method section 2.5. Briefly, two fieldmaps were acquired with reversed phase-encode blips (anterior-to-posterior and posterior-to-anterior), as outlined in Method Section 2.5. These image pairs, which produced distortions in opposing directions, were used to estimate the susceptibility-induced off-resonance field following the method described by Andersson, Skare, and Ashburner (2003), as implemented in FSL (version 6.0.5.1:57b01774).

1.2 Anatomical Data Preprocessing

A total of 1 T1-weighted (T1w) images were found within the input BIDS dataset. The T1-weighted (T1w) image was corrected for intensity non-uniformity (INU) with N4BiasFieldCorrection (Tustison et al. 2010), distributed with ANTs 2.3.3 (Avants et al. 2008, RRID:SCR_004757), and used as T1w-reference throughout the workflow. The T1w-reference was then skull-stripped with a *Nipype* implementation of the antsBrainExtraction.sh workflow (from ANTs), using OASIS30ANTs as target template. Brain tissue segmentation of cerebrospinal fluid (CSF), white-matter (WM) and gray-matter (GM) was performed on the brain-extracted T1w using fast (FSL 6.0.5.1:57b01774, RRID:SCR_002823, Zhang, Brady, and Smith 2001). Brain surfaces were reconstructed using recon-all (FreeSurfer 6.0.1, RRID:SCR_001847, Dale, Fischl,

and Sereno 1999), and the brain mask estimated previously was refined with a custom variation of the method to reconcile ANTs-derived and FreeSurfer-derived segmentations of the cortical gray-matter of Mindboggle (RRID:SCR_002438, Klein et al. 2017). Volume-based spatial normalization to one standard space (MNI152NLin2009cAsym) was performed through nonlinear registration with antsRegistration (ANTs 2.3.3), using brain-extracted versions of both T1w reference and the T1w template. The following template was selected for spatial normalization: *ICBM 152 Nonlinear Asymmetrical template version 2009c* [Fonov et al. (2009), RRID:SCR_008796; TemplateFlow ID: MNI152NLin2009cAsym].

1.3 Functional Data Preprocessing

For each of the 6 BOLD runs found per subject (across all tasks and sessions), the following preprocessing was performed. First, a reference volume and its skull-stripped version were generated by aligning and averaging 5 single-band references (SBRefs). For single-echo dataset, the reference volume is the single-band references (SBRefs). Head-motion parameters with respect to the BOLD reference (transformation matrices, and six corresponding rotation and translation parameters) are estimated before any spatiotemporal filtering using mcflirt (FSL 6.0.5.1:57b01774, Jenkinson et al. 2002). The estimated *fieldmap* was then aligned with rigid-registration to the target EPI (echo-planar imaging) reference run. The field coefficients were mapped on to the reference EPI using the transform. BOLD runs were slice-time corrected to 0.969s (0.5 of slice acquisition range 0s-1.94s) using 3dTshift from AFNI (Cox and Hyde 1997, RRID:SCR_005927). The BOLD reference was then co-registered to the T1w reference using bbregister (FreeSurfer) which implements boundary-based registration (Greve and Fischl 2009). Co-registration was configured with six degrees of freedom. First, a reference volume and its skull-stripped version were generated using a custom methodology of *fMRIPrep*. Several confounding time-series were calculated based on the *preprocessed BOLD*: framewise displacement (FD), DVARS and three region-wise global signals. FD was computed using two formulations following Power (absolute sum of relative motions, Power et al. (2014)) and Jenkinson (relative root mean square displacement between affines, Jenkinson et al. (2002)). FD and DVARS are calculated

for each functional run, both using their implementations in *Nipype* (following the definitions by Power et al. 2014). The three global signals are extracted within the CSF, the WM, and the whole-brain masks. Additionally, a set of physiological regressors were extracted to allow for component-based noise correction (*CompCor*, Behzadi et al. 2007). Principal components are estimated after high-pass filtering the *preprocessed BOLD* time-series (using a discrete cosine filter with 128s cut-off) for the two *CompCor* variants: temporal (tCompCor) and anatomical (aCompCor). tCompCor components are then calculated from the top 2% variable voxels within the brain mask. For aCompCor, three probabilistic masks (CSF, WM and combined CSF+WM) are generated in anatomical space. The implementation differs from that of Behzadi et al. in that instead of eroding the masks by 2 pixels on BOLD space, the aCompCor masks are subtracted a mask of pixels that likely contain a volume fraction of GM. This mask is obtained by dilating a GM mask extracted from the FreeSurfer's *aseg* segmentation, and it ensures components are not extracted from voxels containing a minimal fraction of GM. Finally, these masks are resampled into BOLD space and binarized by thresholding at 0.99 (as in the original implementation). Components are also calculated separately within the WM and CSF masks. For each CompCor decomposition, the *k* components with the largest singular values are retained, such that the retained components' time series are sufficient to explain 50 percent of variance across the nuisance mask (CSF, WM, combined, or temporal). The remaining components are dropped from consideration. The head-motion estimates calculated in the correction step were also placed within the corresponding confounds file. The confound time series derived from head motion estimates and global signals were expanded with the inclusion of temporal derivatives and quadratic terms for each (Satterthwaite et al. 2013). Frames that exceeded a threshold of 0.5 mm FD or 1.5 standardised DVARS were annotated as motion outliers. All resamplings can be performed with *a single interpolation step* by composing all the pertinent transformations (i.e. head-motion transform matrices, susceptibility distortion correction when available, and co-registrations to anatomical and output spaces). Gridded (volumetric) resamplings were performed using antsApplyTransforms (ANTs), configured with Lanczos interpolation to

minimize the smoothing effects of other kernels (Lanczos 1964). Non-gridded (surface) resamplings were performed using mri_vol2surf (FreeSurfer).

Many internal operations of *fMRIPrep* use *Nilearn* 0.8.1 (Abraham et al. 2014, RRID:SCR_001362), mostly within the functional processing workflow. For more details of the pipeline, see the section corresponding to workflows in *fMRIPrep*'s documentation.

2 Impacts of ME-WC and ME-ICA on the Distribution of Statistical Significance

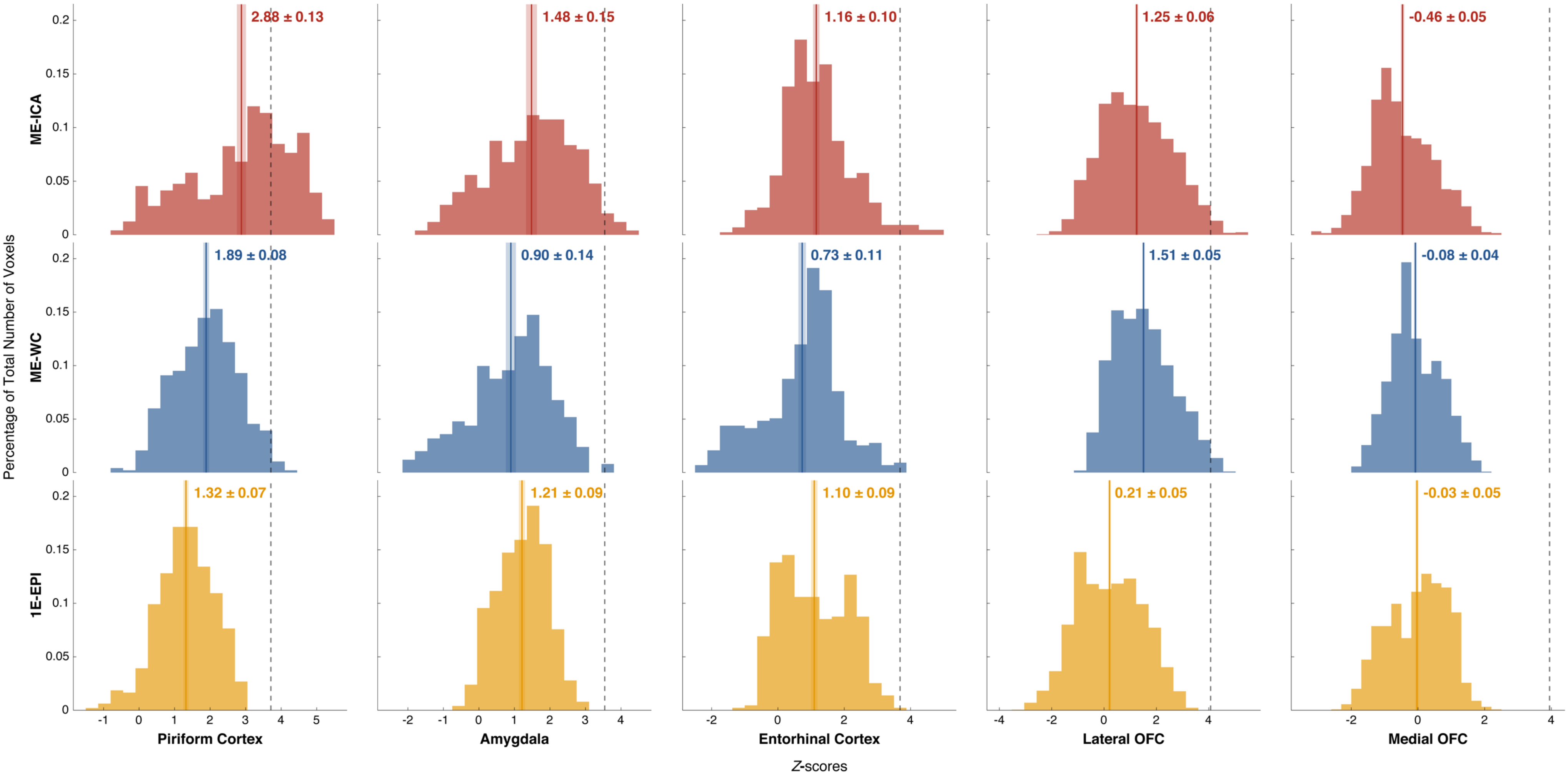

Figure S1. Histograms of Group Level Z-Scores for All ROIs for [Lemon > Control] Contrast. The mean ± 95% confidence interval of the *Z*-scores within each ROI for each acquisition is annotated. The mean is also marked by the solid line surrounded by a shaded area indicating the 95% confidence interval. The dashed line indicates the threshold corresponding to statistical significance ($p < 0.05$, SVC).

In Figure S1, the number of voxels (cluster size) exceeding the statistical significance thresholds (indicated by the dashed lines) and the peak *Z*-scores are higher for ME-WC compared to 1E-EPI. ME-ICA further increases the number of voxels above these thresholds, which is consistent with the results from voxel-wise whole brain analysis (Table 2 and Figure 3b). When examining the mean values and the 95% confidence intervals (CI) of the distribution, we observed that ME-WC has more voxels shifting left past the thresholds, increasing the

suprathreshold cluster size as well as peak *Z*-scores and thus widening the distribution and increasing variance. In other words, ME-WC tends to have a wider CI, indicating larger variance and a broader spread of the distribution.

The significance of the ROI analysis depends on both the mean and the variance of the distribution. In some regions, such as the entorhinal cortex, ME-WC increases the within-ROI variance without increasing the mean, and thus the statistical significance decreases compared to 1E-EPI. We interpret this as meaning that while ME-WC has increased sensitivity to positively correlated BOLD activation, it has also increased the sensitivity to anti-correlated signals.

3 T_2^* Changes Due to Respiration

	p-value	T_2^* [Inhalation] (ms)	T_2^* [Exhalation] (ms)	ΔT_2^* [Inhalation – Exhalation] (ms)
**Piriform Cortex**	0.0109*	54.50 ± 1.48	54.13 ± 1.45	0.36 ± 0.26
**Amygdala**	0.0011**	52.32 ± 1.76	52.12 ± 1.75	0.20 ± 0.11
**Entorhinal Cortex**	0.0004***	33.25 ± 2.48	33.12 ± 2.45	0.13 ± 0.06
**Lateral OFC**	0.0521	33.45 ± 1.09	33.38 ± 1.11	0.06 ± 0.06
**Medial OFC**	0.0334*	24.92 ± 1.08	24.87 ± 1.08	0.05 ± 0.04

Table S1. T_2^* Changes during Inhalation and Exhalation Phase in All Five ROIs. The *p*-values indicate the statistical significance of the two-sample, two-sided *t*-tests comparing T_2^* values estimated from volumes during the inhalation phase with those from the exhalation phase. T_2^* values and their differences (ΔT_2^*) are reported as mean values within the each ROI across all subjects ± 95% confidence intervals.

To investigate T_2^* changes during respiration, we estimated T_2^* maps for each subject at both inhalation and exhalation phases. As described in the Methods section, the respiratory trace was downsampled to match the *TR* of the fMRI acquisition. Inhalation phase volumes are defined as those 0.8 standard deviations above the mean of the downsampled respiratory trace, while exhalation phase volumes are defined as those 0.8 standard deviations below the mean.

We extracted the mean T_2^* values for each subject at both inhalation and exhalation phases and performed two-sample, two-sided *t*-tests to determine if there were statistically significant differences between the two phases. All ROIs, except the lateral orbitofrontal cortices (OFC), showed significant, though subtle, differences, with increased T_2^* values during the inhalation phase, as shown in Table S1. As a result, the weights in the T_2^*-weighted combination method (ME-WC) remain relatively stable across different respiratory phases.

4 Nonexponential Decay of Multi-Echo GE-EPI Signal

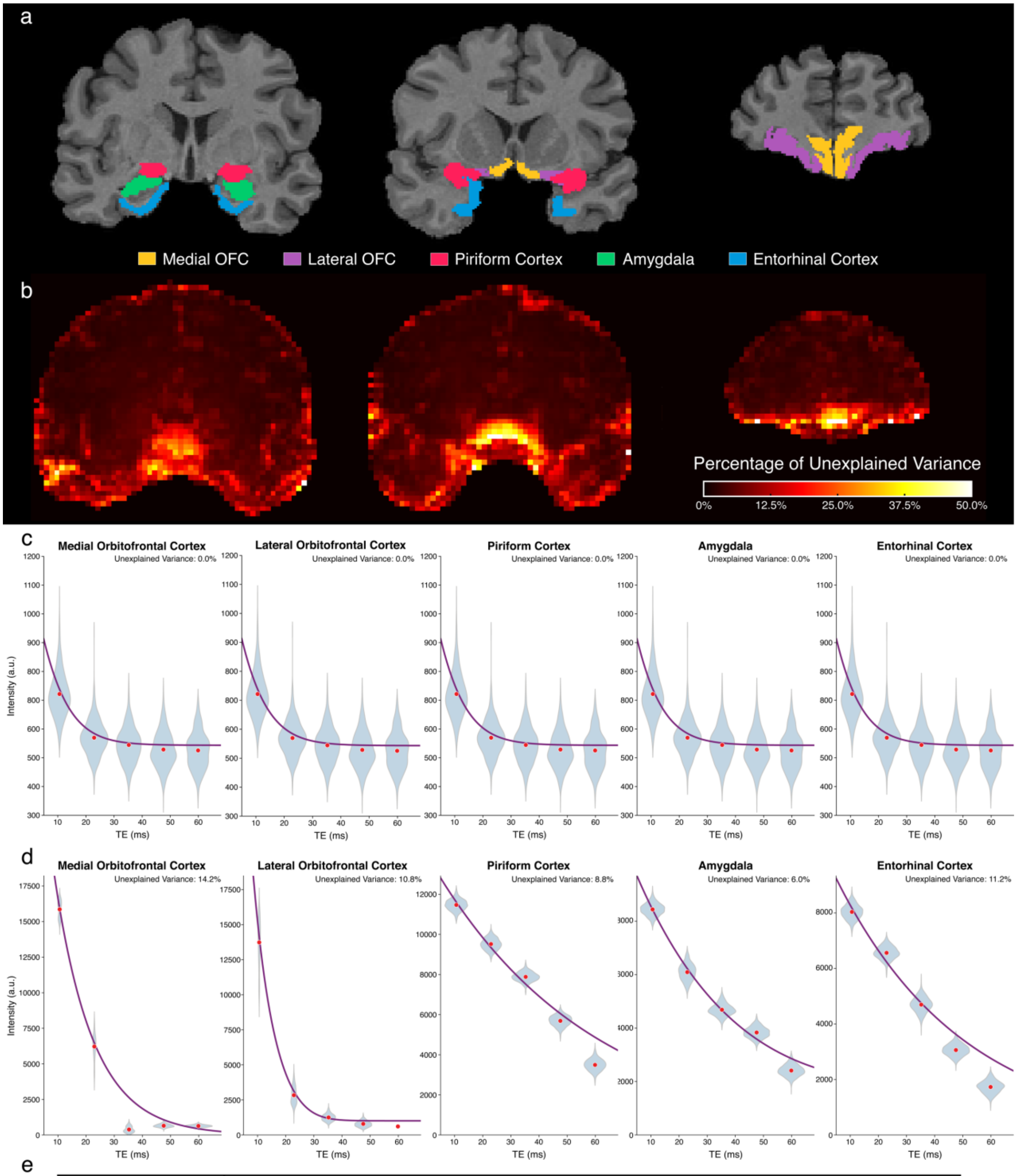

e

	Medial OFC	Lateral OFC	Piriform Cortex	Amygdala	Entorhinal Cortex
**Unexplained Variance** (%)	14.15 ± 0.82	10.84 ± 0.36	8.79 ± 0.60	5.98 ± 0.59	11.11 ± 0.54

Figure S2. Percentage of Unexplained Variance in the Exponential Decay Signal across Different Echo Times for a Single Subject. a) Three representative coronal slices of a single subject with ROIs overlaid on T_1-weighted anatomical images. b) The percentage of unexplained variance map for the representative coronal slices. c) Signal across different echo times for voxels with the minimum unexplained variance in each ROI. The red dots represent the mean signal intensity over 600 measurements (200 volumes per run, 3 runs in total), and the blue violin plots show the distribution of signal intensity. The purple lines indicate the fitted model using the method described in Section 2.7. d) Signal across different echo times for voxels with the unexplained variance closest to the mean unexplained variance within each ROI. The red dots, blue violin plots, and purple lines represent the mean signal intensity, distributions, and fitted model, respectively. e) The mean ± 95% confidence interval of unexplained variance for each ROI.

Theoretically, the BOLD signal under GE-EPI should exhibit an exponential decay across echo times. To examine deviations from this expected exponential decay, we calculated the voxel-wise percentage of unexplained variance. This calculation was performed by dividing the root mean square (RMS) of the residuals by the RMS of the observed signal, as shown in Figure S3. Regions with high susceptibility, such as the medial and lateral orbitofrontal cortices (OFC) and the entorhinal cortex, demonstrated a more pronounced nonexponential decay.

5 Supplementary Figures and Tables

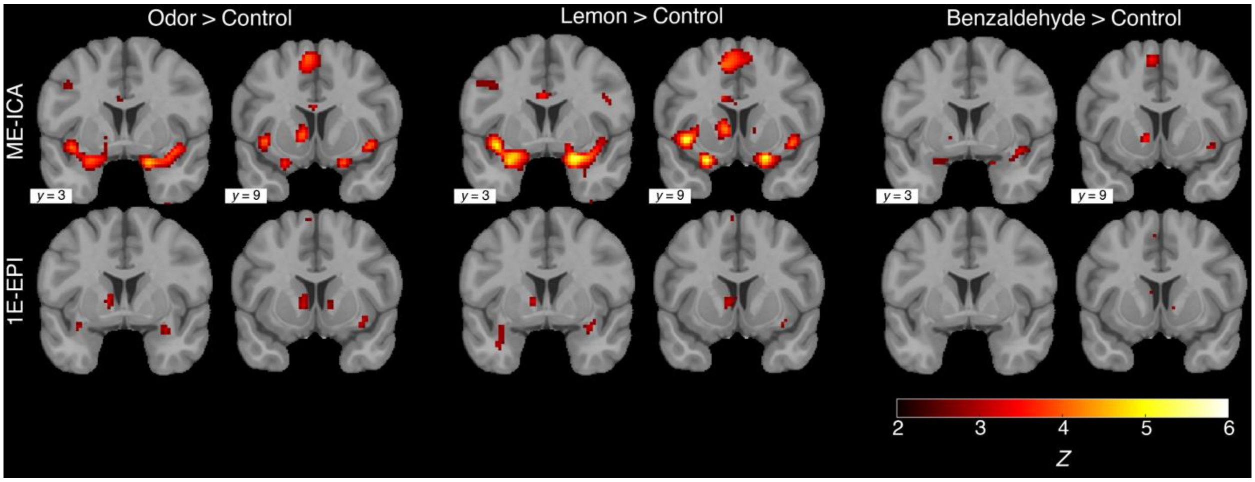

Figure S3. Comparison of Activation Maps among Different Contrasts for Olfactory-Related Tasks in Both 1E-EPI and ME-EPI. Two representative coronal slices of activation maps for all three contrasts ([Odor > Control], [Benzaldehyde > Control], [Lemon > Control]) using ME-EPI acquisition with ME-ICA denoising methods (ME-ICA) and using single-echo fMRI (1E-EPI) acquisition (p < 0.001, uncorrected).

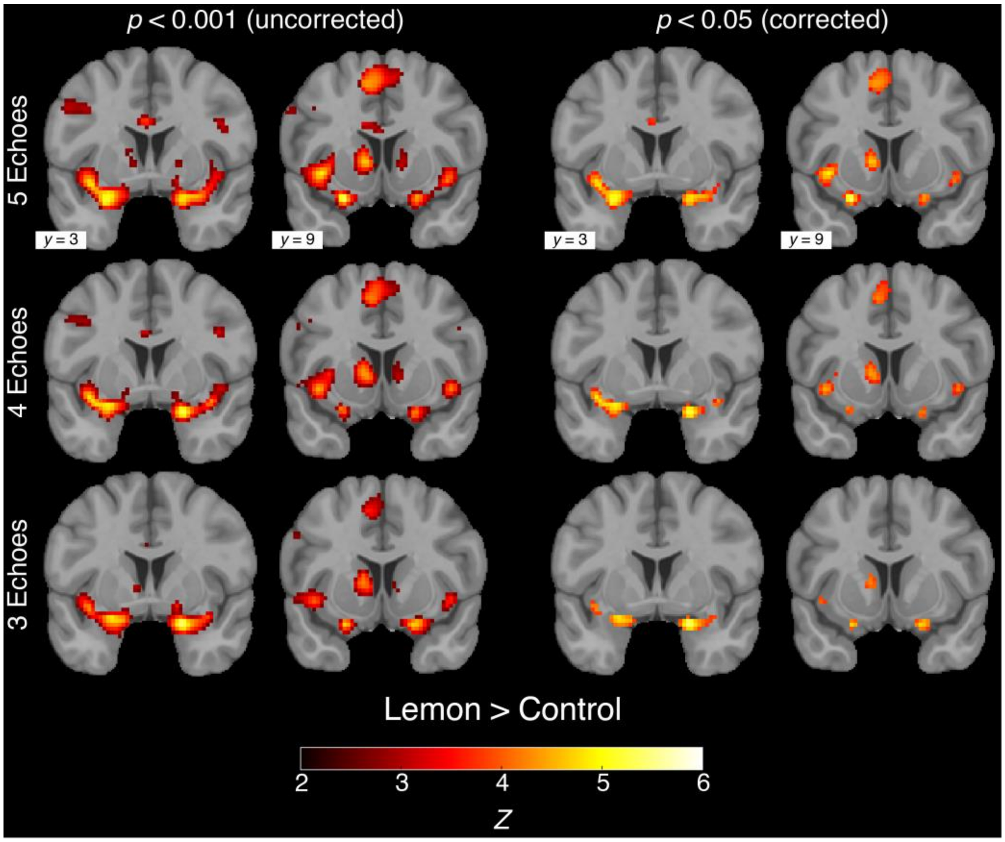

Figure S4. Comparison of Activation Maps for ME-EPI Acquisition with ME-ICA Denoising among Different Number of Echoes for Olfactory-Related Tasks. Two representative coronal slices of activation maps for [Lemon > Control] using ME-EPI acquisition with ME-ICA denoising methods (ME-ICA) with first 3, 4 and all 5 echoes. The left two columns show the maps without multiple comparison correction ($p < 0.001$) while the right two columns show the maps corrected for multiple comparison ($p < 0.05$, FDR corrected).

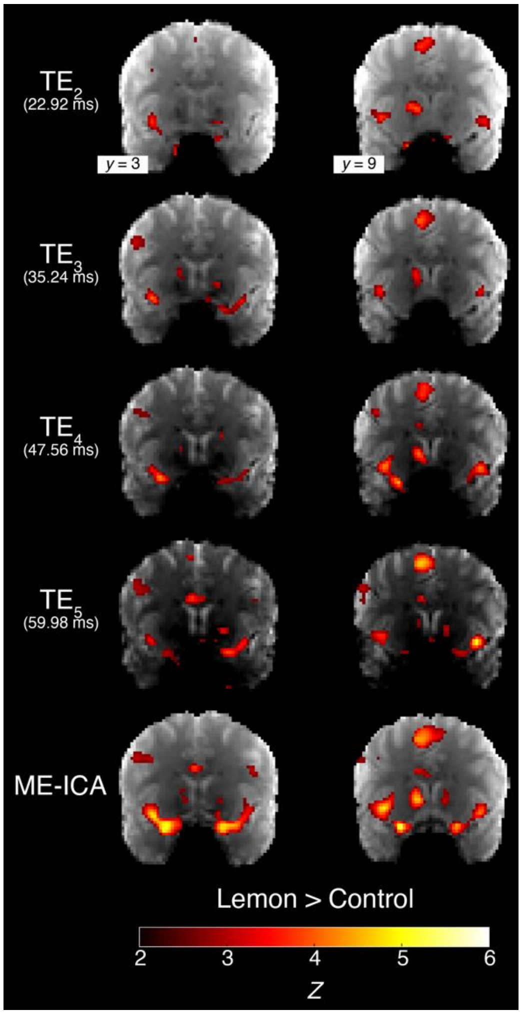

Figure S5. Comparison of Activation Maps When Analyzing Individual Echoes from ME-ICA Separately for Olfactory-Related Tasks. Two representative coronal slices of activation maps for [Lemon > Control] when analyzing individual echoes from ME-EPI acquisition with ME-ICA denoising methods (ME-ICA) separately on the group level ($p < 0.05$, uncorrected). The activation maps are overlaid on the mean image of a single subject across one run, registered to MNI template space, to illustrate signal dropout across different echoes.

	Piriform Cortex	Amygdala	Entorhinal Cortex	Orbitofrontal Cortex
**TE_1** (10.60 ms)	1.945	1.010	0.756	1.205
**TE_2** (22.92 ms)	2.383	1.957	2.183	1.605
**TE_3** (35.24 ms)	3.457	1.583	1.789	1.724
**TE_4** (47.56 ms)	3.819	1.355	0.973	1.835
**TE_5** (59.98 ms)	3.693	2.604	1.265	1.705
**1E-EPI** (22.00 ms)	2.479	1.972	2.152	-0.279

Table S2. Z-Scores of ROI Analyses for Each Olfactory-related ROI under the Contrast [Lemon > Control] When Analyzing 5 Individual Echoes from ME-ICA Separately.

	Medial OFC	Lateral OFC	Piriform Cortex	Amygdala	Entorhinal Cortex
**Unmodified T_2^* Estimation** (ms)	21.9 ± 0.6	30.2 ± 0.6	45.8 ± 1.1	45.6 ± 1.1	29.0 ± 1.0
**Modified T_2^* Estimation** (ms)	13.0 ± 0.2	21.8 ± 0.2	41.3 ± 1.1	42.2 ±1.0	22.2 ± 0.3

Table S3. Comparison of Estimated T_2^* Values between Unmodified and Modified Fitting Methods for All ROIs within a Single Subject. Unmodified T_2^* estimation method fits Eq. (1) for each voxel. Modified T_2^* estimation method fits Eq. (3) instead.

6 Supplementary References

Abraham, Alexandre, Fabian Pedregosa, Michael Eickenberg, Philippe Gervais, Andreas Mueller, Jean Kossaifi, Alexandre Gramfort, Bertrand Thirion, and Gael Varoquaux. 2014. "Machine Learning for Neuroimaging with Scikit-Learn." *Frontiers in Neuroinformatics* 8. https://doi.org/10.3389/fninf.2014.00014.

Andersson, Jesper L. R., Stefan Skare, and John Ashburner. 2003. "How to Correct Susceptibility Distortions in Spin-Echo Echo-Planar Images: Application to Diffusion Tensor Imaging." *NeuroImage* 20 (2): 870–88. https://doi.org/10.1016/S1053-8119(03)00336-7.

Avants, B. B., C. L. Epstein, M. Grossman, and J. C. Gee. 2008. "Symmetric Diffeomorphic Image Registration with Cross-Correlation: Evaluating Automated Labeling of Elderly and Neurodegenerative Brain." *Medical Image Analysis* 12 (1): 26–41. https://doi.org/10.1016/j.media.2007.06.004.

Behzadi, Yashar, Khaled Restom, Joy Liau, and Thomas T. Liu. 2007. "A Component Based Noise Correction Method (CompCor) for BOLD and Perfusion Based fMRI." *NeuroImage* 37 (1): 90–101. https://doi.org/10.1016/j.neuroimage.2007.04.042.

Cox, Robert W., and James S. Hyde. 1997. "Software Tools for Analysis and Visualization of fMRI Data." *NMR in Biomedicine* 10 (4-5): 171–78. https://doi.org/10.1002/(SICI)1099-1492(199706/08)10:4/5<171::AID-NBM453>3.0.CO;2-L.

Dale, Anders M., Bruce Fischl, and Martin I. Sereno. 1999. "Cortical Surface-Based Analysis: I. Segmentation and Surface Reconstruction." *NeuroImage* 9 (2): 179–94. https://doi.org/10.1006/nimg.1998.0395.

Esteban, Oscar, Ross Blair, Christopher J. Markiewicz, Shoshana L. Berleant, Craig Moodie, Feilong Ma, Ayse Ilkay Isik, et al. 2018. "fMRIPrep." *Software*. https://doi.org/10.5281/zenodo.852659.

Esteban, Oscar, Christopher Markiewicz, Ross W Blair, Craig Moodie, Ayse Ilkay Isik, Asier Erramuzpe Aliaga, James Kent, et al. 2018. “fMRIPrep: A Robust Preprocessing Pipeline for Functional MRI.” *Nature Methods*. https://doi.org/10.1038/s41592-018-0235-4.

Fonov, VS, AC Evans, RC McKinstry, CR Almli, and DL Collins. 2009. “Unbiased Nonlinear Average Age-Appropriate Brain Templates from Birth to Adulthood.” *NeuroImage* 47, Supplement 1: S102. https://doi.org/10.1016/S1053-8119(09)70884-5.

Gorgolewski, K., C. D. Burns, C. Madison, D. Clark, Y. O. Halchenko, M. L. Waskom, and S. Ghosh. 2011. “Nipype: A Flexible, Lightweight and Extensible Neuroimaging Data Processing Framework in Python.” *Frontiers in Neuroinformatics* 5: 13. https://doi.org/10.3389/fninf.2011.00013.

Gorgolewski, Krzysztof J., Oscar Esteban, Christopher J. Markiewicz, Erik Ziegler, David Gage Ellis, Michael Philipp Notter, Dorota Jarecka, et al. 2018. “Nipype.” *Software*. https://doi.org/10.5281/zenodo.596855.

Greve, Douglas N, and Bruce Fischl. 2009. “Accurate and Robust Brain Image Alignment Using Boundary-Based Registration.” *NeuroImage* 48 (1): 63–72. https://doi.org/10.1016/j.neuroimage.2009.06.060.

Jenkinson, Mark, Peter Bannister, Michael Brady, and Stephen Smith. 2002. “Improved Optimization for the Robust and Accurate Linear Registration and Motion Correction of Brain Images.” *NeuroImage* 17 (2): 825–41. https://doi.org/10.1006/nimg.2002.1132.

Klein, Arno, Satrajit S. Ghosh, Forrest S. Bao, Joachim Giard, Yrjö Häme, Eliezer Stavsky, Noah Lee, et al. 2017. “Mindboggling Morphometry of Human Brains.” *PLOS Computational Biology* 13 (2): e1005350. https://doi.org/10.1371/journal.pcbi.1005350.

Lanczos, C. 1964. “Evaluation of Noisy Data.” *Journal of the Society for Industrial and Applied Mathematics Series B Numerical Analysis* 1 (1): 76–85. https://doi.org/10.1137/0701007.

Posse, Stefan, Stefan Wiese, Daniel Gembris, Klaus Mathiak, Christoph Kessler, Maria-Liisa Grosse-Ruyken, Barbara Elghahwagi, Todd Richards, Stephen R. Dager, and Valerij G. Kiselev. 1999. “Enhancement of BOLD-Contrast Sensitivity by Single-Shot Multi-Echo Functional MR Imaging.” *Magnetic Resonance in Medicine* 42 (1): 87–97. https://doi.org/10.1002/(SICI)1522-2594(199907)42:1<87::AID-MRM13>3.0.CO;2-O.

Power, Jonathan D., Anish Mitra, Timothy O. Laumann, Abraham Z. Snyder, Bradley L. Schlaggar, and Steven E. Petersen. 2014. “Methods to Detect, Characterize, and Remove Motion Artifact in Resting State fMRI.” *NeuroImage* 84 (Supplement C): 320–41. https://doi.org/10.1016/j.neuroimage.2013.08.048.

Satterthwaite, Theodore D., Mark A. Elliott, Raphael T. Gerraty, Kosha Ruparel, James Loughead, Monica E. Calkins, Simon B. Eickhoff, et al. 2013. “An improved framework for confound regression and filtering for control of motion artifact in the preprocessing of resting-state functional connectivity data.” *NeuroImage* 64 (1): 240–56. https://doi.org/10.1016/j.neuroimage.2012.08.052.

Tustison, N. J., B. B. Avants, P. A. Cook, Y. Zheng, A. Egan, P. A. Yushkevich, and J. C. Gee. 2010. “N4itk: Improved N3 Bias Correction.” *IEEE Transactions on Medical Imaging* 29 (6): 1310–20. https://doi.org/10.1109/TMI.2010.2046908.

Zhang, Y., M. Brady, and S. Smith. 2001. “Segmentation of Brain MR Images Through a Hidden Markov Random Field Model and the Expectation-Maximization Algorithm.” *IEEE Transactions on Medical Imaging* 20 (1): 45–57. https://doi.org/10.1109/42.906424
